# Safety and efficacy of complementary and alternative medicine in the treatment of autism spectrum disorder: A protocol for systematic review and meta-analysis

**DOI:** 10.1097/MD.0000000000023128

**Published:** 2020-11-06

**Authors:** Biqin Shuai, Hongjiao Jin, Yong Lin, Renrong Duan, Ning Zhao, Zhu Li, Jiao Mao, Yan Luo, Mengyu Shi

**Affiliations:** The First People's Hospital of Zunyi (The Third Affiliated Hospital of Zunyi Medical University), Huichuan District, Zunyi, Guizhou 563000, China.

**Keywords:** autism spectrum disorder, complementary and alternative medicine, protocol, systematic review

## Abstract

**Introduction:**

The purpose of this study is to evaluate the efficacy and safety of complementary and alternative medicine in the treatment of autism spectrum disorder.

**Methods and analysis:**

We will electronically search Pubmed, Medline, Embase, Web of Science, the Cochrane Central Register of Controlled Trial, China National Knowledge Infrastructure, China Biomedical Literature Database, China Science Journal Database, and Wan-fang Database from their inception. Also, we will manually retrieve other resources, including reference lists of identified publications, conference articles, and gray literature. The clinical randomized controlled trials or quasi-randomized controlled trials related to complementary and alternative medicine treating autism spectrum disorder will be included in the study. The language is limited to Chinese and English. Research selection, data extraction, and research quality assessment will be independently completed by 2 researchers. Data were synthesized by using a fixed-effect model or random-effect model depend on the heterogeneity test. The Childhood Autism Rating Scale (CARS) and Autism Behavior Checklist (ABC) scores will be the primary outcomes. The scores of the Autism Treatment Evaluation Checklist and the Ritvo-Freeman Real Life Rating Scale will also be assessed as secondary outcomes. RevMan V.5.3 statistical software will be used for meta-analysis, and the level of evidence will be assessed by Grading of Recommendations Assessment, Development, and Evaluation (GRADE). Continuous data will be expressed in the form of weighted mean difference or standardized mean difference with 95% confidence intervals (CIs), whereas dichotomous data will be expressed in the form of relative risk with 95% CIs.

**Ethics and dissemination:**

The protocol of this systematic review does not require ethical approval because it does not involve humans. We will publish this article in peer-reviewed journals and presented at relevant conferences.

**Systematic review registration:**

OSF Registries, DOI: 10.17605/OSF.IO/ HA97R (https://osf.io/ha97r)

## 1 Introduction

Similar to other neurodevelopmental disabilities, autism spectrum disorder (ASD) is considered lacking satisfactory cure and requiring long-term management.^[1]^ The prevalence of ASD was reported raising in the last decades, ranged of 6.5 to 6.6 per 1000 or approximately 1 in 160 children are suffering from ASD.^[2–4]^ Regardless of intellectual developing, ASD children remain within the spectrum as adults and reporting problems with their living, working, social abilities and mental health.^[5,6]^

To date, no medication is proven to be effective in treating core symptoms of ASD, varies of approaches investigated the use of anti-psychotics, stimulants and nonstimulants, antidepressants, (GABA)ergic and cholinergic agents, and oxytocin, among others in ASD, the limited evidences unsupported pharmacotherapy in children with ASD, and side-effects with long-term use can be burdensome.^[7]^

Despite of their limited evidence and potential adverse effects, the use of complementary and alternative medicine (CAM) is popular in children with ASD.^[8]^ Among children with ASD, use of CAM ranges from 28% to 51%, with lifetime use as high as 71% in United States, and 46% in Germany.^[8,9]^ CAM treatments are used for a variety of symptoms in children with ASD, including core symptoms of ASD, concentration, relaxation, gastrointestinal symptoms, sleep disturbance, communication, sensory issues, seizures, and for general health.^[10]^ Melatonin, omega-3 fatty acids, methyl B12, oxytocin, vitamin supplementation, ginkgo biloba, acupuncture, hyperbaric oxygen therapy, and chelation therapy are common CAM forms applied on ASD children. These different forms of CAM were administrated for various symptoms in ASD; most parents selected CAM for their concern of side-effects from pharmacotherapy, and they also reported some benefits from CAM, despite of lacking evidences of these CAM treatments.^[11–14]^ Therefore, to investigate the safety and efficacy of CAM in the treatment of ASD, we plan to conduct this systematic review (SR) and meta-analysis.

## 2 Methods

The protocol has been registered on OSF as Registration DOI: 10.17605/OSF.IO/HA97R (https://osf.io/ha97r). The protocol follows the Preferred Reporting Items for Systematic Reviews and Meta-Analyses Protocols (PRISMA-P) 2015 statement guidelines.^[15]^ We will report the changes in the full review if necessary.

### 2.1 Inclusion and exclusion criteria for study selection

#### 2.1.1 Inclusion criteria

This study will include randomized controlled trials (RCTs) of complementary and alternative medicine treatment for autism spectrum disorder patients, whether using blind method or allocation concealment method, including those using a quasi-random method such as alternate allocation or allocation by birth date. We included both parallel and cross-over studies. The language of the trials to be included should be Chinese or English.

#### 2.1.2 Exclusion criteria

Following studies would be excluded: case reports and reviews, literature not in English or Chinese language, and clinical research studies that compared different kinds of CAM.

### 2.2 Types of participants

We would include patients with ASD under the age of 18 years, regardless of sex or race, diagnosed by standard criteria such as the Diagnostic and Statistical Manual of Mental Disorders (DSM) or the International Classification of Diseases (ICD). We accept diagnoses by assessment tools such as the Autism Diagnostic Observation Scale (ADOS), Autism Diagnostic Interview Revised (ADI-R), Childhood Autism Rating Scale (CARS), Chinese Classification of Mental Disorder (CCMD), and other validated tools. Studies on ASD were included even if they did not refer to the diagnostic criteria.

### 2.3 Types of interventions and comparators

CAM therapies for treating ASD include modified/special diets, vitamins/minerals, food supplements, acupuncture, and Chinese medicine. These interventions can be used alone or in combination. Controlled interventions included control groups with no treatment, sham/placebo groups, or other conventional treatments.

### 2.4 Types of outcome measures

#### 2.4.1 Primary outcomes

We select the score of Childhood Autism Rating Scale (CARS) and autism behavior checklist (ABC) as primary outcomes.

#### 2.4.2 Secondary outcomes

We also care about the scores of the Autism Treatment Evaluation Checklist, and the Ritvo-Freeman Real Life Rating Scale; meanwhile, the social interaction skills, communication ability, or stereotypy, language ability, and cognitive function would be taken into consideration.

## 3 Data sources

### 3.1 Electronic searches

Following databases will be searched: PubMed, Web of Science, the Cochrane Central Register of Controlled Trials, AMED, MEDLINE, EMBASE, Cochrane Library, China National Knowledge Infrastructure (CNKI), Wanfang data, Chinese Scientific Journals Database (VIP), and China biomedical literature database (CBM). We will select the eligible studies published up to August 31, 2020. The search terms used in the SR are as follows: complementary and alternative medicine, autism spectrum disorder, neurodevelopmental disorder, melatonin, vitamin, Omega-3 fatty acids, acupuncture, hyperbaric oxygen therapy, chelation therapy, among others.

We will not apply any language, population, or national restrictions. The specific search strategy will be (taking PubMed as an example) listed on Table 1. Similar search strategy will be applied to other electronic databases.

**Table 1:** Search strategy sample of PubMed.

Number	Searches
#1	randomized controlled trial[MeSH Terms]
#2	autism spectrum disorder[MeSH Terms]
#3	autism spectrum disorder[Text Word]
#4	neurodevelopmental disorder[MeSH Terms]
#5	neurodevelopmental disorder[Text Word]
#6	or/#2–5
#7	complementary and alternative medicine[MeSH Terms]
#8	acupuncture[MeSH Terms]
#9	melatonin[MeSH Terms]
#10	L-Carnosine[MeSH Terms]
#11	N-acetylcysteine[MeSH Terms]
#12	Omega-3 fatty acids[MeSH Terms]
#13	vitamin[MeSH Terms]
#14	tetrahydrobiopterin[MeSH Terms]
#15	hyperbaric oxygen therapy[MeSH Terms]
#16	chelation therapy[MeSH Terms]
#17	or/#7–6
#18	#1and#6and#17

We will identify relevant randomized controlled trials and the selected studies will be analyzed according to the Cochrane Handbook.

### 3.2 Searching other resources

**Figure 1:**
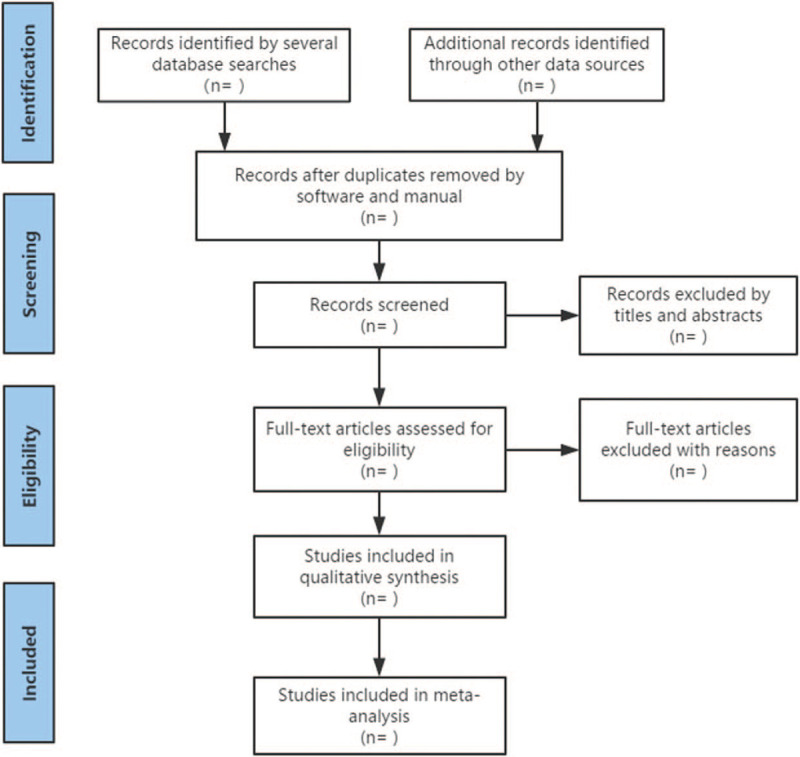
The research flow chart.

We also retrieve manual-related documents, such as replacing and supplementing some reference documents, medical textbooks, clinical laboratory manuals, and the World Health Organization International Registry of Clinical Trials. At the same time, we will contact experts and authors in this field to obtain important information that cannot be found in the search. The research flow chart is shown in Figure 1.

## 4 Data collection and analysis

### 4.1 Selection of studies

Two independent researchers (SB and JH) will assess the full-text articles from the search results independently against the inclusion and exclusion criteria. Discrepancies will be discussed and resolved by consensus with a third author (DR).

### 4.2 Data extraction and management

The following information will be extracted from each study: research number, data extractor, date of data extraction, general situation of the study, research methodology, research population, baseline comparability, interventions, main outcome indicators, secondary outcome indicators, combined drug use, adverse reactions or complications, among others. For those with questions or incomplete information, we will try to contact the author to obtain information before deciding whether to include it.

### 4.3 Assessment of the reporting quality and risk of bias

Two of the authors (SB and JH) individually assessed the risk of bias using assessments included in the study were evaluated in the Cochrane System Evaluator's Manual for RCT quality evaluation criteria. Assessing the risk of bias: random sequence generation; allocation concealment; blinding of participants and personnel; blinding of outcome assessment; incomplete outcome data; selective outcome reporting; other bias. Every domain was classified as high risk of bias, low risk of bias or unclear risk of bias. Any arising difference was resolved by discussion.

### 4.4 Measures of a treatment effect

We will measure continuous data with mean difference (MD) or standard MD for the therapeutic effect with 95% CIs. For dichotomous data, risk ratios (RR) with 95% CIs will be calculated.

### 4.5 Management of missing data

To obtain the missing data, we will contact the corresponding author. If no response will be obtained, we will analyze only the available data and describe the reason and impact of this exclusion in the paper.

### 4.6 Assessment of a reporting bias

Publication bias will be explored through funnel plot analysis. GRADE profiler 3.6 is used to evaluate the quality of evidence. The specific contents include: limitations of research, inconsistency of research results, indirect evidence, inadequate accuracy, publication bias. Finally, the quality of evidence is divided into 4 levels: high-level evidence, intermediate evidence, low-level evidence, and very low-level evidence.

### 4.7 Assessment of heterogeneity

All literature will use *I*^2^ value of the *χ*^2^ test (a = 0.1) to determine the heterogeneity. When *I*^2^ ≤50%, it is considered acceptable. When *I*^2^ > 50%, subgroup analysis should be performed to identify potential causes and record them.

### 4.8 Data synthesis and grading of quality of evidence

RevMan 5.3 software was used for statistical analysis of data. RR was used for binary variables and MD was used for continuous variables. Heterogeneity analysis will be conducted by heterogeneity test, *P* and *I*^2^ represent the size of heterogeneity among multiple studies. When *P* is >0.1 and *I*^2^ is <50%, it suggests heterogeneity is small, and on the contrary, it suggests heterogeneity is large. Heterogeneity is mainly handled by subgroup analysis. Sensitivity analysis is used to test the reliability of the overall effect.

### 4.9 Subgroup analysis

When the heterogeneity test results are heterogeneous, we will conduct subgroup analysis to explore the possible causes of heterogeneity. The effects of different types of acupuncture therapy including design scheme, severity of illness, age, sex, and mild or severe AP were analyzed. We will also delete low-quality and/or medium-quality studies to check the robustness of the results.

### 4.10 Sensitivity analysis

Sensitivity analysis will be used to test the quality of the research contained in the sampled documents. The stability of the conclusions can be tested by re-analyzing the conclusions by inputting missing data and changing the type of research.

### 4.11 Ethics and dissemination

The results of the system review will be published in peer-reviewed journals, disseminated at relevant meetings, or disseminated in peer-reviewed publications, and we use aggregated published data to exclude individual patient data, so ethical approval, and informed consent is not required.

## 5 Discussion

CAM treatments are popular in the intervention of ASD. Evidences showed different forms of CAM might bring ASD children benefit in different aspects.

Studies showed the efficacy of melatonin in treating sleep disturbance on ASD children and adolescents, it was reported to be well tolerated and safe.^[16,17]^ Omega-3 fatty acids have been examined to be a potential treatment for ASD, specific for the associated symptoms of hyperactivity, although most studies failed to show statistical significance in improving either autism core symptoms or hyperactivity, omega-3 fatty acids were showed well-tolerated.^[18–21]^ As for vitamin D supplementation, a study found significant improvement on ASD children for CARS and ABC scores, and the improvements were more pronounced in children younger than 3 years.^[22]^ As a major form of Chinese medicine, from clinical trials and SRs, acupuncture is reported to improve various developmental and behavioral aspects of children with ASD, it's safe but children experienced pain and cried during the treatment.^[23–26]^ Tetrahydrobiopterin is an important cofactor in the biosynthesis of catecholamines and serotonin, and in the treatment of ASD, it is reported to significantly improve social interaction subscale of the CARS.^[27]^l-Carnosine is a dipeptide known for its antioxidant properties and proposed enhancement of γ-aminobutyric acid function in the brain, with possible anticonvulsive effects, it was showed to significantly improve CARS score on ASD children.^[28]^ N-acetylcysteine (NAC) is an antioxidant with involvement in extracellular glutamate modulation, it seemed to be well-tolerated, and it might bring some benefits to ASD children for the associated symptom of irritability.^[29]^ However, in treating ASD, some commonly used CAM treatments are reported ineffective, including methyl B12, oxytocin, ginkgo biloba, secretin, hyperbaric oxygen therapy, and chelation therapy.^[30–38]^

As a conclusion, CAM is widely used in the management of ASD, although evidences for their safety and efficacy are still in lack. We would like to conduct this SR and meta-analysis to further investigate CAM's clinical practice on ASD. However, there are some potential limitations of our study. First, differences of methodologic quality in the trials may cause significant heterogeneity. In addition, due to the limitations of language ability, we would only search literature in English and Chinese; this may lead to the potential risk of ignoring essential literature.

## Author contributions

Biqin Shuai and Hongjiao Jin are co-first authors and contribute equally.

**Conceptualization:** Biqin Shuai, Yong Lin.

**Data curation:** Hongjiao Jin.

**Formal analysis:** Biqin Shuai.

**Funding acquisition:** Yong Lin.

**Methodology:** Yong Lin, Ning Zhao.

**Project administration:** Hongjiao Jin, Yong Lin.

**Software:** Renrong Duan.

**Supervision:** Yong Lin, Biqin Shuai

**Validation:** Zhu Li.

**Visualization:** Jiao Mao.

**Writing – original draft:** Biqin Shuai, Hongjiao Jin.

**Writing – review & editing:** Shenghua Liu, Mengyu Shi, Yan Luo.
